# Insights from distribution dynamics inform strategies to conserve a dhole *Cuon alpinus* metapopulation in India

**DOI:** 10.1038/s41598-019-39293-0

**Published:** 2019-02-28

**Authors:** Arjun Srivathsa, K. Ullas Karanth, N. Samba Kumar, Madan K. Oli

**Affiliations:** 1 School of Natural Resources and Environment, University of Florida, Gainesville, FL USA; 2 Department of Wildlife Ecology and Conservation, University of Florida, Gainesville, FL USA; 3 Wildlife Conservation Society-India, Bengaluru, India; 4 Centre for Wildlife Studies, Bengaluru, India; 5 Wildlife Conservation Society, Global Conservation Program, New York, NY USA; 6 National Centre for Biological Sciences, Bengaluru, India

## Abstract

Most large carnivore populations currently occur in heterogeneous landscapes, with source populations embedded in a matrix of human-dominated habitats. Understanding changes in distribution of endangered carnivores is critical for prioritizing and implementing conservation strategies. We examined distribution and dynamics of a dhole *Cuon alpinus* metapopulation, first in 2007 and subsequently in 2015, based on indirect sign surveys across 37, 000sq. km of India’s Western Ghats. Predicted dhole occupancy declined from 0.62 (95% CI: 0.58–0.66) in 2007 to 0.54 (95% CI: 0.50–0.58) in 2015. Occupancy was associated with abundance of primary prey species and anthropogenic disturbance. Local extinction appeared to be influenced by forest cover loss, and offset by protected reserves; colonization was influenced by occupancy in neighbouring sites. Perturbation analysis indicated that occupancy was more sensitive to local extinction within reserves and to colonization in sites abutting reserves. The Western Ghats could serve as a stronghold for the endangered dhole, provided future colonizations are facilitated through habitat consolidation beyond reserve boundaries, and local extinctions are prevented by increasing protection efforts within select reserves. We advocate for wildlife managers to adopt a landscape-based approach and periodic monitoring to ensure persistence of the dhole metapopulation in Western Ghats, and in other critical conservation regions across the species’ geographic range.

## Introduction

Rapid anthropogenic changes in land-use patterns and fragmentation of natural habitats over time has resulted in spatial structuring of several animal populations across heterogeneous landscapes^1,2^. Such systems now comprise fragmented or otherwise spatially structured populations with source populations typically restricted to protected areas, embedded in a matrix of human-dominated land-uses that serve as sinks^3–5^. These populations are more likely to persist in landscapes with multiple high quality habitat patches capable of supporting reproductive populations, connected by functional corridors that can facilitate movement, rather than in insular habitats^6^. Continued loss and fragmentation of natural habitats that support such metapopulations, however, poses constraints for individuals’ movement between habitat patches, reduces demographic and genetic connectivity among sub-populations, and subsequently results in population declines and local extinctions^7,8^.

Temporal variations in distribution and abundance, resource availability, and landscape features can cause changes in patch occupancy of metapopulations. These changes can be modeled and monitored through estimation of vital rates like colonizations and local extinctions within habitat patches where species’ metapopulations occur^5,9^. Ecologists, conservation biologists, and wildlife managers have sought to understand such spatio-temporal trends in colonization and local extinction patterns for the past few decades through application of stochastic patch occupancy models^9,10^. These metrics may be important when determining the ability of habitat patches to sustain spatially structured populations of threatened or endangered species.

Despite their ecological importance, charismatic appeal, and decades of conservation efforts, several species of large carnivores have witnessed global population declines^11^. A recent assessment by Wolf and Ripple^12^ suggests that the world’s large carnivores have experienced drastic range contractions. Species such as the red wolf *Canis rufus*, tiger *Panthera tigris*, and African wild dog *Lycaon pictus* have range contractions of >90% from their former geographic range. Currently, large carnivores are found in 34% of the world’s land area in highly fragmented landscapes^12^, facilitated by resource availability and affected adversely by anthropogenic threats^13^. Among the large carnivores, conserving wild canids is particularly difficult because they are more frequently prone to conflict with humans, face consequent persecution, and are susceptible to spread of diseases from domestic animals in shared landscapes^14^.

The Asiatic wild dog or dhole *Cuon alpinus* is a wild canid that primarily inhabits forested areas, and is an apex social carnivore in tropical forests of south and southeast Asia^15–17^. Globally dholes have disappeared from ~82% of their former range^12^. Although a thorough population assessment is currently lacking, India perhaps supports the highest number of dholes. While their occurrence is mostly limited to forested wildlife reserves, presence of dhole packs has also been reported from unprotected secondary forests, multi-use forest fragments, and agro-forest plantations abutting protected reserves^18–21^. The Western Ghats landscape in India harbors a dhole metapopulation, with potential population sources in some protected reserves, and the unprotected forest fragments between them probably serving as population sinks or movement corridors^19^. Following rapid economic growth and infrastructure development in the region, there have been extensive changes in land-use patterns, leading to loss of potential dhole habitats^22^. An assessment of spatio-temporal changes in dhole distribution is therefore pertinent for understanding patterns of colonizations and local extinctions.

In this study, we examined patterns and dynamics of dhole distribution in the Western Ghats of India from 2007 to 2015. Specifically, we (1) estimated the proportion of landscape currently occupied by dholes, (2) assessed changes in dhole occupancy patterns over time- through comparisons with previous estimates of their distribution in the landscape, and (3) examined factors that influence probabilities of colonizations and local extinctions. We predicted *a priori* that dhole distribution would be influenced by a combination of ecological, anthropogenic and management factors; changes in dhole occupancy would be driven by habitat quality, protection status of dhole habitats, and colonization and local extinction patterns rooted in metapopulation theory. We then determined the sensitivity of dhole occupancy to changes in local extinctions and colonizations, and the corresponding predictor variables. Based on the dynamic occupancy model parameters and sensitivity analysis, we propose management interventions for conserving the metapopulation.

## Methods

### Study Area

**Figure 1:**
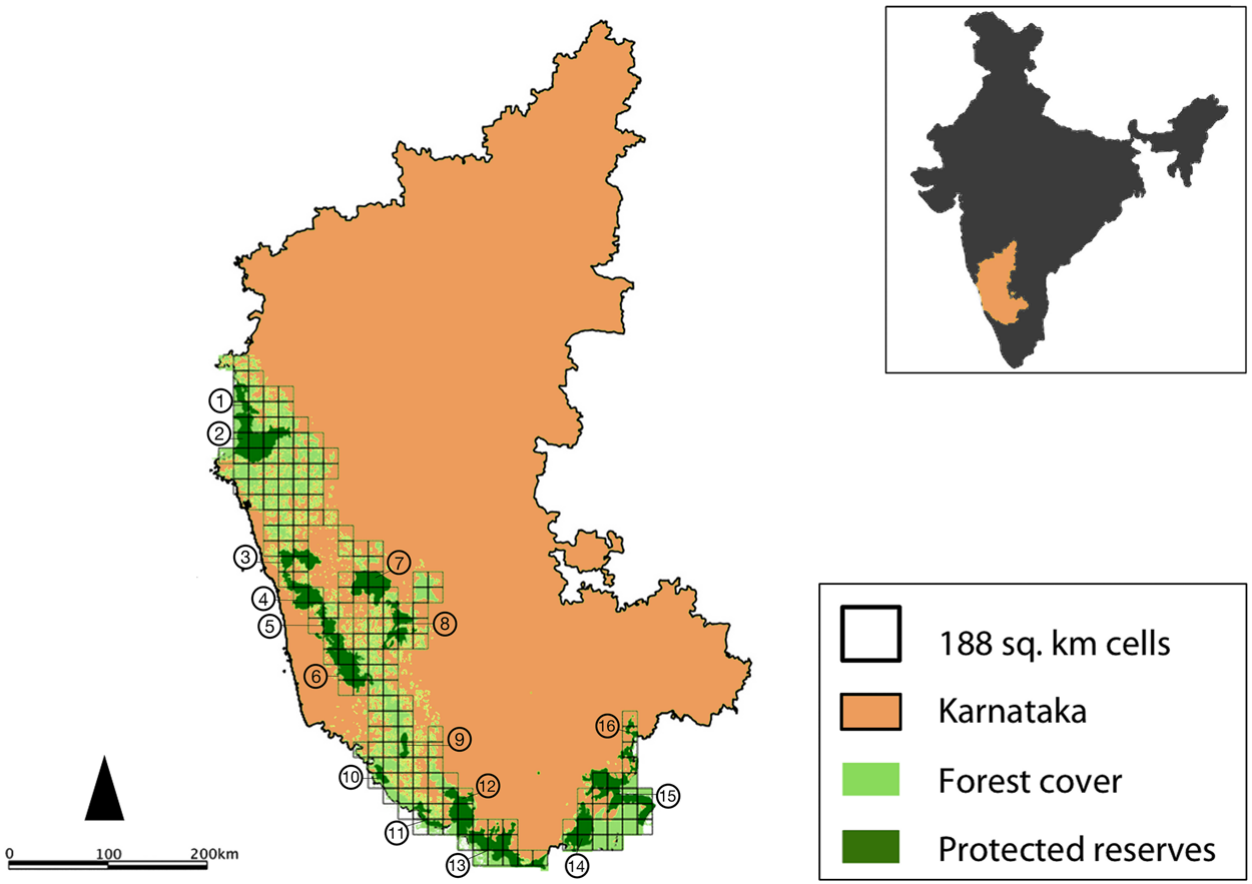
Study area in the Western Ghats of Karnataka, India. The map shows forest cover and protected wildlife reserves, with superimposition of 197 grid-cells measuring 188sq. km each. Protected reserves: (1) Bhimgad, (2) Kali, (3) Sharavati, (4) Mookambika, (5) Someshwara, (6) Kudremukh, (7) Shettihalli, (8) Bhadra, (9) Pushpagiri, (10) Talakaveri, (11) Brahmagiri, (12) Nagarahole, (13) Bandipur, (14) Biligiri Rangaswamy Temple, (15) Cauvery-MM Hills, (16) Bannerghatta. Inset: location of Karnataka State in India.

The Western Ghats of India is a global biodiversity hotspot with 58 wildlife reserves nested within a much larger multi-use landscape of about 160,000sq. km. The elevation varies from 300 m to 2700 m, with higher altitudes restricted to the central ridge of the mountain range. The landscape is spread across the States of Gujarat, Maharashtra, Goa, Karnataka, Tamil Nadu and Kerala. The western and eastern slopes of the Ghats consist of semi-evergreen, tropical moist-deciduous, and dry-deciduous forests with substantial anthropogenic modifications, creating a heterogeneous vegetation matrix^23^. Most of the forested areas are within protected reserves, with large expanses of production landscapes (such as coffee, areca nut, tea, and rubber) around the reserves^23^. We conducted this study in the Western Ghats within the State of Karnataka (Fig. 1). The *c*. 20,000sq. km of forests support a diverse assemblage of wild ungulates such as the chital *Axis axis*, four-horned antelope *Tertracerus quadricornis*, gaur *Bos gaurus*, mouse deer *Moschiola indica*, muntjac *Muntiacus muntjak*, sambar *Rusa unicolor* and wild pig *Sus scrofa*. The landscape also has populations of dhole and its co-predators, the tiger and the leopard *Panthera pardus*^24^. There are 16 protected reserves within the study landscape, encompassing an area of around 8700sq. km.

### Field surveys

Metapopulation models typically treat habitat patches as spatial units, where individual patches are identified as source or sink areas based on the occupancy status and population vital rates of the species^4,5,9,25^. Due to logistical constraints and the relatively large geographic extent of our study, we used a grid-based design and identified clusters of grid-cells (cells with reserves) as plausible population sources and the larger unprotected forest landscape as potential sink areas. We carried out indirect sign surveys across *c*. 37, 000sq. km area of Karnataka’s Western Ghats, first in 2006–2007 and subsequently in 2014–2015. The study area was gridded with square cells of 188sq. km each (Fig. 1). The size of the cells was chosen such that each sample unit was larger than the maximum home range size of dhole packs in the area (~85sq. km)^26^. The data were collected following an occupancy sampling design implemented for surveys of tigers in the same landscape^27,28^. Surveys were conducted by multiple teams, each consisting of three members. Each team included one experienced research assistant, one local field assistant and one citizen volunteer. The research assistants with multiple years of field experience trained local field assistants and volunteers. All surveyors were trained in mammal sign recognition using photographs of scats, spoors, measurement of tracks/stride lengths and identification of secondary signs. Surveyors recorded data only for signs that could be unambiguously attributed to the species. When in doubt, surveyors clicked pictures using point-and-shoot cameras and cross-checked with senior research staff at the base camp. Although species misclassification is a possibility, we belive this would be an extremely small proportion of the dataset.

Field surveys were carried out across 206 grid-cells during the dry seasons of 2006 and 2007 (henceforth, ‘2007 survey’), maintaining uniform detection conditions, with an assumption that dhole distribution did not change over the larger landscape in this period. The sampling effort per grid-cell was standardized such that a cell with 100% forest cover would receive ~45 km of walk effort (roughly the length of two diagonals, to yield adequate spatial coverage) and the effort would decrease proportionally with decrease in forest cover (e.g., a cell with 50% cover would have ~22 km walk effort). Data related to dhole signs were recorded as either ‘1’ (detected) or ‘0’ (undetected) on successive 1-km trail/road segments. The number of spatial replicates sampled per cell in 2007 ranged from 2 to 42 (survey effort being proportional to the percentage of forest cover in each grid-cell). The second survey was undertaken between 2014 and 2015 (henceforth, ‘2015 survey’) across 208 grid-cells, during the same months as in the 2007 surveys. The number of spatial replicates sampled per grid-cell in the 2015 survey ranged from 4 to 49. For this study, we used data from 197 grid-cells that were common between the two surveys (2007 and 2015; Fig. 1). We hereafter refer to grid-cells as ‘sites’.

### Analytical methods

The dynamic occupancy model or the ‘multi-season’ model described by MacKenzie *et al*.^29^ allows for examining changes in species distributions over time, while explicitly modeling colonization/local extinction probabilities and accounting for imperfect detection. The model is structured such that probabilities of occupancy at time *t* are a function of site-specific colonization/extinction probabilities and occupancy status from the previous time step *t* - 1^30^. We applied a variant of this model^31^, which is ideally suited for surveys conducted along linear habitats (such as forest roads/trails) where detections tend to be spatially correlated^27^. The model has four key parameters: ψ^1^ (probability of presence at a site in season 1), *p*_*t*_ (probability of detection in a spatial replicate, given presence in the site and presence in the replicate), γ (probability of colonization) and ε (probability of local extinction). Parameters θ^0^ and θ^1^ represent the extent of spatial dependence between detections along sequential replicates. In this model, probability of presence at a site in season 2 (ψ^2^) is a derived parameter^30^, obtained as:

$${{\rm{\psi }}}^{2}={{\rm{\psi }}}^{1}(1-{\rm{\varepsilon }})+(1-{{\rm{\psi }}}^{1}){\rm{\gamma }}$$

### Covariates for modeling occupancy patterns and dynamics

Based on current knowledge of dhole ecology in the landscape^15,19,21^, we used a combination of ground-based and remotely sensed covariates pertaining to ecological (relative abundance of all prey and relative abundance of principal prey, i.e., chital) and anthropogenic (relative abundance of livestock) factors to examine their distribution patterns. Data corresponding to ground-based covariates were collected during field surveys (additional details on covariates are provided in Table 1). In modeling ψ^1^ and *p*_*t*_, we used the same covariate combination from the top-ranked model presented in Srivathsa *et al*.^19^.

**Table 1:** Descriptions of ecological, anthropogenic and management-related covariates used to model probabilities of dhole occupancy (ψ^1^), colonization (γ), local extinction (ε) and detection (*p*_*t*_) probabilities in the Western Ghats of Karnataka, India; *a priori* predictions of the direction of covariate effect, and data sources.

Parameter	Covariates	Covariate description and *a priori* prediction	Source
ψ^1^	Chital abundance (cht)	Current knowledge of dhole diet in the region suggests that chital or spotted deer is its primary prey species. Relative abundance for each site calculated as the proportion of sampled replicates that had signs of chital presence. Predicted effect: Positive	Data collected during field surveys
ψ^1^	Livestock abundance (lvs)	Srivathsa *et al*.^19^ report that livestock presence inside forests best represent extent of anthropogenic intrusions in dhole habitat. Relative abundance of livestock calculated as the proportion of sampled replicates that had signs of livestock presence. Predicted effect: Negative	Data collected during field surveys
*p*_*t*_	Abundance of prey (apr)	Chital, sambar, muntjac, gaur and wild pigs constitute >90% of biomass in dhole diet. Relative abundance of all prey species in each site was calculated as the proportion of sampled replicates that had signs of at least one of the five species. Predicted effect: Positive	Data were collected during field surveys
γ, ε	Change in forest cover (fch)	Change in forest cover in each site measured as a log-transformed ratio of % cover in 2015 and % cover in 2007. Predicted effect: Positive for γ and Negative for ε; sites with increase in forest cover are likely to be colonized. Decrease in forest cover would be associated with higher local extinction probabilities.	Forest cover layers obtained from Indian Institute of Remote Sensing, Govt. of India
γ, ε	Reserve presence (rpres)	Either 0 (reserve absent) or 1(reserve present) for each site. Predicted effect: Positive for γ and Negative for ε; Local extinction would be higher in sites that do not have reserves	Geo-spatial maps of reserves obtained from the State Forest Department
γ, ε	Reserve area (rarea)	Area under designated protected reserves in each site. Predicted effect: Positive for γ and Negative for ε; colonization would be higher in sites with larger extent of protection, local extinction would be lower in sites with larger reserve area	Geo-spatial maps of reserves obtained from the State Forest Department
γ, ε	Neighborhood effect (nbr)	Calculated for each site as the sum of estimated occupancy probabilities (in 2007) of its first-order neighbor sites. Predicted effect: Positive for γ and Negative for ε	Estimates derived from Srivathsa *et al*.^19^

Dynamic parameters γ (colonization) and ε (local extinction) may be influenced by ecological variables, management-related factors, and neighborhood effects. Here, we define colonization as the probability that a site not occupied by dholes in 2007 is occupied in 2015, and local extinction as the probability that a site occupied by dholes in 2007 is no longer occupied in 2015. We modeled these parameters as functions of change in forest cover (proportional change; calculated as log-ratios of forest cover in 2015 and 2007), and presence (0 or 1) and area of protected reserves in each site (Table 1). In the context of reduced forest cover in the landscape^22^, we predicted that local extinction probabilities would be driven by reduction in forest cover, and offset by presence and extent of protected reserves; probabilities of local extinction would therefore be higher outside the protected area network. We expected probabilities of colonization to be higher in sites that have protected reserves, assuming that there was no significant decline in the overall population of dholes and their prey species within reserves (over the eight-year period between the two surveys).

Neighborhood effects are often observed in metapopulations across heterogeneous landscapes, where colonization of a site in time *t* + 1 is influenced by the occupancy status of its neighbor sites at time *t*^32,33^. Expecting a similar process for dhole populations in this landscape, we modeled site-specific colonization/extinction probabilities as functions of the occupancy state of their neighbor sites from the previous time step. We calculated neighborhood effect for each site as a sum of estimated (unconditional) occupancy probabilities of its first order neighbors (up to eight sites around the focal site) in 2007; these estimates were obtained from Srivathsa *et al*.^19^ because they provided information from nine additional sites that were excluded from the present study. We make two assumptions in our treatment of the neighborhood effect in this manner; (1) given the large size of the sites in relation to the known home-range size of dhole packs, colonizations would be through neighborhood effects rather than directional movement, and (2) in the absence of any evidence for long-distance dispersal by dholes, colonization/local extinction effects would be most evident in first-order neighbor sites rather than across distant habitat patches (or across reserves) in the landscape. Detailed covariate descriptions and data sources are provided in Table 1.

We first checked for cross-correlations between pairs of covariates to avoid multi-collinearity issues while fitting the models (Pearson’s *r* range for continuous covariates: −0.38–0.26). We refrained from using reserve presence and forest cover change in the same model because they were strongly correlated (pseudo R^2^ = 0.32; *r* = 0.59). We scaled and normalized all the non-categorical covariates such that the mean = 0 and σ = 1. This ensured that estimated β-coefficients represented changes in logit(*Y*) for a 1σ change in the covariate (where *Y* is a model parameter: ψ, γ, ε, or *p*_*t*_). Model fits were compared and ranked based on Akaike’s Information Criterion (AIC). We conducted occupancy analyses under a maximum likelihood framework, implemented in program PRESENCE version 11.9.

### Perturbation analysis

The sensitivity of a species’ occupancy to changes in colonization/local extinction (extent of change in occupancy as a result of small changes in colonization/local extinction probabilities) is important for understanding occupancy dynamics of populations^34^. Inferences drawn from such analyses can also contribute towards determining management actions^30,35^. Sensitivity analysis requires estimation of long-term equilibrium occupancy (ψ^*^), which is similar to the asymptotic population growth rate for populations (λ), calculated as:

$${{\rm{\psi }}}^{\ast }=\frac{{\rm{\gamma }}}{{\rm{\gamma }}+{\rm{\varepsilon }}}$$

We calculated sensitivity of ψ^*^ to colonization $({S}_{{\rm{\gamma }}})$ and local extinction $({S}_{{\rm{\varepsilon }}})$ as:

$$\,{S}_{{\rm{\gamma }}}=\frac{\partial {{\rm{\psi }}}^{\ast }}{\partial {\rm{\gamma }}}=\frac{{\rm{\varepsilon }}}{{({\rm{\gamma }}+{\rm{\varepsilon }})}^{2}}$$

$${S}_{{\rm{\varepsilon }}}=\frac{\partial {{\rm{\psi }}}^{\ast }}{\partial {\rm{\varepsilon }}}=-\,\frac{{\rm{\gamma }}}{{({\rm{\gamma }}+{\rm{\varepsilon }})}^{2}}$$

We used absolute values of the two sensitivity metrics so that the magnitudes could be directly compared. We first calculated ψ^*^, *S*_γ_ and *S*_ε_ for a fully constrained model [i.e., γ(.), ε(.) formulation]. Next, we estimated the three parameters for within and outside reserves [γ(*rpres*), ε(*rpres*)], with an expectation that ψ^*^ would be more sensitive to colonization outside reserves, and more sensitive to local extinction within reserves. Third, we examined the role of reserve area [a time-invariant, continuous predictor; γ(*rarea*), ε(*rarea*)], to obtain a gradient of sensitivities for the landscape. We chose locations with the highest values (the upper 25% quartile) for colonization and local extinction based on site-specific sensitivities to recommend management actions.

## Results

The 2007 survey involved 2021 person-days and 4109 km of walk effort, resulting in a total encounter of 267 dhole signs (201 scats and 66 tracks) along 154 1-km replicates. The subsequent 2015 survey involved 2280 person days and 4808 km of walk effort, and yielded 251 dhole signs (190 scats and 61 tracks) along 146 1-km replicates. Based on naïve estimates of occupancy (without accounting for partial detectability), dhole signs were detected in 35% of the sites in 2007 and 30% of the sites in 2015.

### Patterns and dynamics of dhole occupancy

We compared 20 candidate covariate models to examine factors influencing occupancy, colonization, local extinction, and detectability of dholes in the landscape (tested against a model with intercept-only formulation of parameters). Based on the intercept-only formulation, parameter estimates were: ψ^1^ (s.e.) = 0.65 (0.07), γ (s.e.) = 0.28 (0.15) and ε (s.e.) = 0.30 (0.10). Models where ψ^1^ was described as a function of the relative abundance of chital and livestock (based on Srivathsa *et al*.^19^) performed better than those where ψ^1^ was held constant (intercept-only model). We modeled detectability as a function of relative abundance of all prey species, while allowing it to vary by season (*p*_*t*_^1^ ≠ *p*_*t*_^2^). We also tested for this seasonal effect on the spatial dependence parameters (θ^0^, θ^1^ in 2007 ≠ θ^0^, θ^1^ in 2015), but these models received less support compared to the model where there was no seasonal effect. Similarly, models that included ecological, anthropogenic and neighborhood effects on the dynamic parameters had better support compared to models where we treated colonization and local extinction to be constant across sites (Table 2 and Supplementary Table S1).

**Table 2:** Model comparisons to identify covariates influencing dhole occupancy in 2007 (ψ^1^), colonization (γ), local extinction (ε) and detection (*p*_*t*_) probabilities in the Western Ghats of Karnataka.

Model	Model description	AIC	ΔAIC	AIC weight	Model likelihood	K	Deviance
M1	ψ^1^(*cht* + *lvs*), θ^0^(.), θ^1^(.), γ(*nbr*), ε(*fch*), *p*_*t*_(*ssn* + *apr*)	2318.25	0	0.24	1	14	2290.25
M2	ψ^1^(*cht* + *lvs*), θ^0^(.), θ^1^(.), γ(*nbr*), ε(*rpres*), *p*_*t*_(*ssn* + *apr*)	2319.57	1.32	0.12	0.51	14	2291.57
M3	ψ^1^(*cht* + *lvs*), θ^0^(.), θ^1^(.), γ(.), ε(*fch*), *p*_*t*_(*ssn* + *apr*)	2319.74	1.49	0.11	0.47	13	2293.74
M4	ψ^1^(*cht* + *lvs*), θ^0^(.), θ^1^(.), γ(*nbr* + *rpres*), ε(*fch*), *p*_*t*_(*ssn* + *apr*)	2320.06	1.81	0.10	0.40	15	2290.06
M5	ψ^1^(*cht* + *lvs*), θ^0^(.), θ^1^(.), γ(*nbr* + *rarea*), ε(*fch*), *p*_*t*_(*ssn* + *apr*)	2320.07	1.82	0.09	0.40	15	2290.07
M20	ψ^1^(.), θ^0^(.), θ^1^(.), γ(.), ε(.), *p*_*t*_(.)	2363.71	45.46	0	0	7	2349.71

The table includes estimates for the top five models from the candidate set (ΔAIC < 2) and the intercept-only model (M20). Full list of candidate models are provided in Supplementary Information (S1). cht- chital abundance; lvs- livestock abundance; nbr- neighborhood effect; fch- forest cover change; apr- all prey abundance; rpres- reserve presence; rarea- reserve area; ssn- season; K- number of parameters.

**Figure 2:**
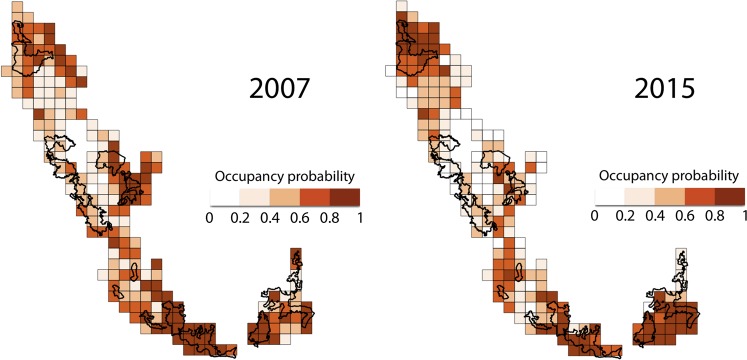
Patterns of dhole distribution in the Western Ghats of Karnataka, India. (**a**) Estimates of site-specific occupancy probabilities in 2007 where the average occupancy is 62% and (**b**) estimated occupancy probabilities in 2015 where the average occupancy in the landscape is 54%.

**Figure 3:**
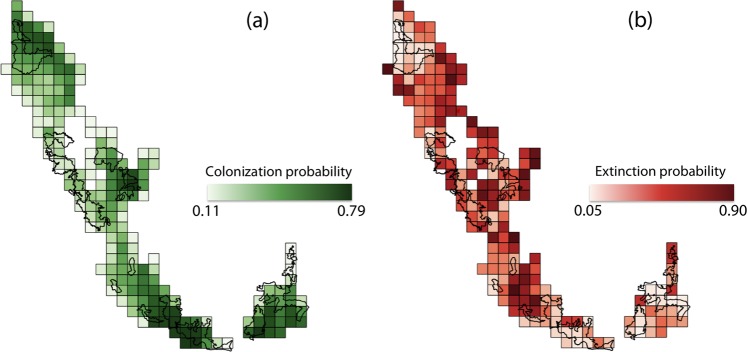
Patterns of dhole occupancy dynamics in the Western Ghats of Karnataka, India. (**a**) colonizations and (**b**) local extinctions of dholes in Karnataka’s Western Ghats, between 2007 and 2015. Site-specific values are based on model-averaged estimates of the dynamic parameters.

Because several models received comparable support (Table 2), we model-averaged across all candidate models to obtain estimates of the key parameters (ψ^1^, γ, ε, *p*_*t*_^1^ and *p*_*t*_^2^). We did so by multiplying parameter estimates from each model with the corresponding model weights and summing across the weighted parameters. This allowed us to account for comparable support offered by multiple candidate models. Dholes occupied 62% of the landscape in 2007 [ψ^1^ (s.e.; 95% CI) = 0.62 (0.02; 0.58–0.66); Fig. 2]. Detectability varied across seasons, but remained somewhat similar in 2007 and 2015 [*p*_*t*_^1^ (s.e.) = 0.35 (0.004); *p*_*t*_^2^ (s.e.) = 0.30 (0.01)]. The magnitude of difference between estimates of θ^0^ and θ^1^ provided evidence for spatial dependence in detections along forest roads/trails [θ^0^ (s.e.) = 0.07 (0.01) and θ^1^ (s.e.) = 0.61 (0.001)]. Estimates of the dynamic parameters indicated that model-averaged colonization probability was higher than local extinction probability in the landscape [γ (s.e.) = 0.43 (0.01); ε (s.e.) = 0.38 (0.02)], although both these parameters showed high site-specific variation (Fig. 3). Dhole occupancy in 2015 (derived parameter ψ^2^) declined to 54% [ψ^2^ (s.e.; 95% CI) = 0.54 (0.02; 0.50–0.58); Fig. 2].

### Determinants of occupancy, colonization and local extinction

**Figure 4:**
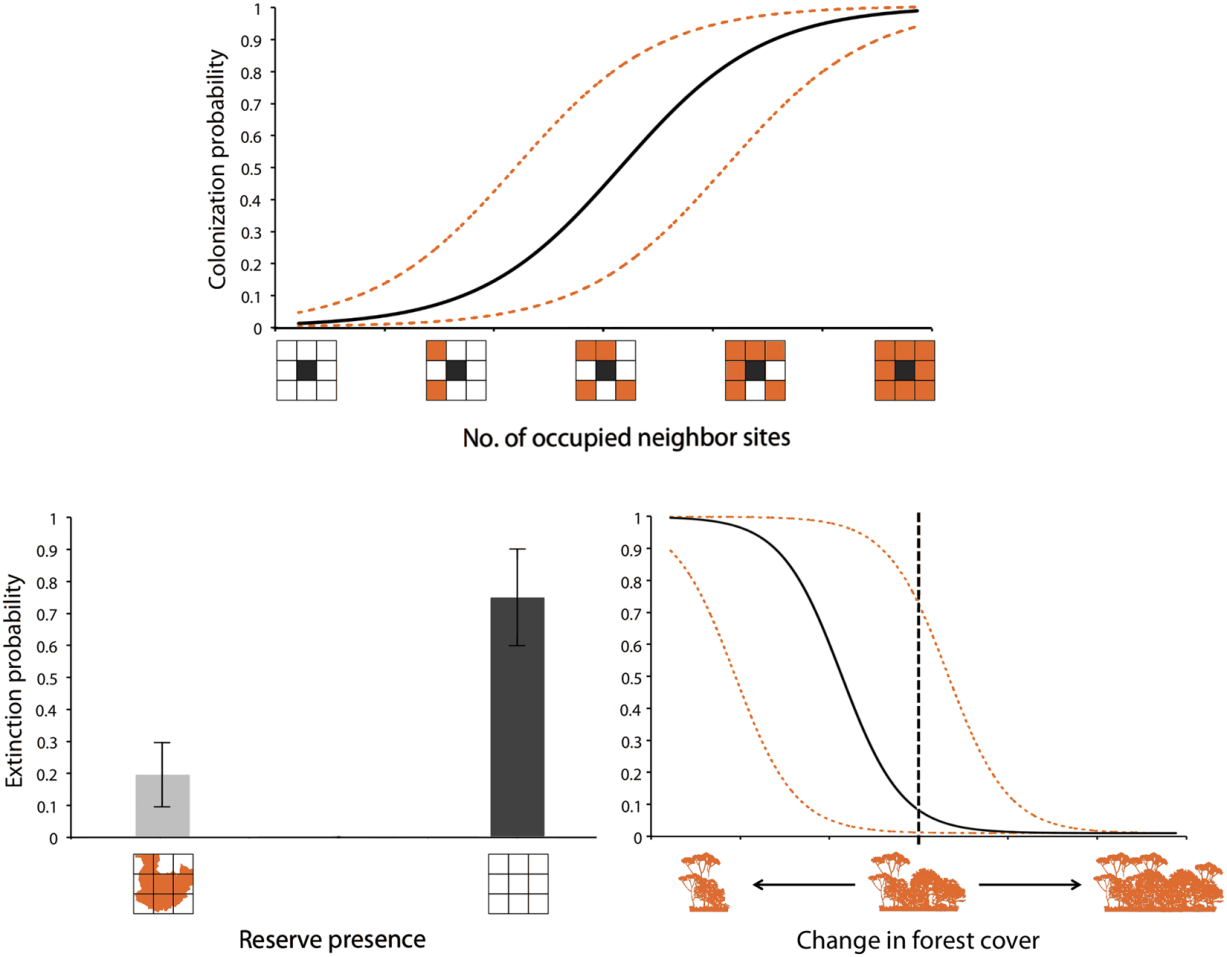
Correlates of occupancy dynamics. Top panel: Relationship between number of occupied sites (neighborhood effect) on colonization probability for dholes based on model M1 (x-axis range: 0.81–7.89); dotted lines are 95% confidence intervals. Bottom panel: Difference in average local extinction probabilities for dholes between sites with and without reserves, based on model M2 (left). Relationship between estimated probabilities of local extinctions with change in forest cover during the eight-year period, based on model M1 (right). Change in forest cover calculated as log-ratios of forest habitat proportion between 2007 and 2015 (x-axis range: −1.23–2.44); dotted lines are 95% confidence intervals and vertical line represents ‘no change’ in forest cover.

We note that for most covariates the direction of effect was indicative rather than conclusive, since the 95% confidence intervals for their respective β-coefficients straddled zero. We found that relative abundance of chital was positively associated with dhole presence [β_*cht*_ (s.e.) = 1.63 (0.899) from the top-ranked model], while anthropogenic disturbance– measured as relative abundance of livestock– had a negative effect [β_*lvs*_ (s.e.) = −0.89 (0.46)]. Relative abundance of all prey species was positively associated with detectability in both the surveys [*p*_*t*_^1^: β_*apr*_ (s.e.) = 0.31 (0.16); *p*_*t*_^2^: β_*apr*_ (s.e.) = 0.60 (0.16)]. Neighborhood effect was positively associated with colonization probability [β_*nbr*_ (s.e.) = 1.76 (1.25) from the top-ranked model]; i.e., a site was more likely to be colonized in 2015 if its first-order neighbor sites collectively had higher occupancy in 2007. While models that included colonization probability as functions of presence and extent of reserve area received some support (based on AIC), their corresponding standard error values were large (Tables 2 and 3). Local extinction probability was negatively associated with change in forest cover [β_*fch*_ (s.e.) = −6.66 (3.97); Fig. 4]; decrease in forest cover increased extinction probabilities. Local extinction was also negatively associated with presence of protected reserves [β_*rpres*_ (s.e.) = −2.5 (0.96); Fig. 4]. Estimated β-coefficients for all covariates from models that received the highest support (ΔAIC < 2) are provided in Table 3.

**Table 3:** Estimates of β-coefficients (standard errors in parentheses) for individual covariates associated with probabilities of dhole occupancy in 2007 (ψ^1^), colonization (γ) and local extinction (ε) probabilities, detectability in 2007 (*p*_*t*_^*1*^; season 1) and detectability in 2015 (*p*_*t*_^*2*^; season 2), in the Western Ghats of Karnataka.

	ψ^1^			γ				ε			*p*_*t*_^*1*^		*p*_*t*_^*2*^	
	β_int_	β_*cht*_	β_*lvs*_	β_int_	β_*nbr*_	β_*rpres*_	β_*rarea*_	β_int_	β_*fch*_	β_*rpres*_	β_int_	β_*apr*_	β_int_	β_*apr*_
M1	1.06 (0.64)	1.63 (0.89)	−0.89 (0.46)	−0.29 (0.75)	1.76 (1.25)	—	—	−2.56 (1.79)	−6.66 (3.97)	—	−0.61 (0.31)	0.30 (0.16)	−0.96 (0.28)	0.59 (0.16)
M2	0.89 (0.57)	1.59 (0.80)	−0.72 (0.48)	−0.10 (0.67)	1.34 (0.95)	—	—	1.09 (0.80)	—	−2.51 (0.96)	−0.57 (0.31)	0.30 (0.16)	−0.88 (0.29)	0.61 (0.16)
M3	1.40 (0.73)	2.09 (1.07)	−0.96 (0.47)	−0.68 (0.63)	—	—	—	−2.87 (2.28)	−7.26 (4.94)	—	−0.63 (0.30)	0.31 (0.15)	−0.95 (0.28)	0.59 (0.16)
M4	0.98 (0.64)	1.59 (0.88)	−0.80 (0.46)	−0.54 (0.90)	1.59 (1.18)	0.60 (1.30)	—	−2.47 (1.67)	−6.51 (3.76)	—	−0.59 (0.31)	0.31 (0.16)	−0.97 (0.28)	0.60 (0.16)
M5	1.18 (0.73)	1.56 (0.86)	−1.08 (0.66)	−0.49 (1.02)	2.66 (3.50)	—	−0.79 (1.90)	−2.97 (2.95)	−7.26 (6.12)	—	−0.63 (0.30)	0.32 (0.16)	−0.97 (0.28)	0.59 (0.16)
**Σw**	**—**	**1**	**1**	**—**	**0.69**	**0.18**	**0.10**	**—**	**0.63**	**0.28**	**—**	**1**	**—**	**1**

The table includes estimates for the top five models from the candidate set (ΔAIC < 2) and summed AIC weights across all models (Σw) for covariates. int-intercept; cht- chital abundance; lvs- livestock abundance; nbr- neighborhood effect; fch- forest cover change; apr- all prey abundance; rpres- reserve presence; rarea- reserve area.

### Sensitivity to colonization and local extinction

**Figure 5:**
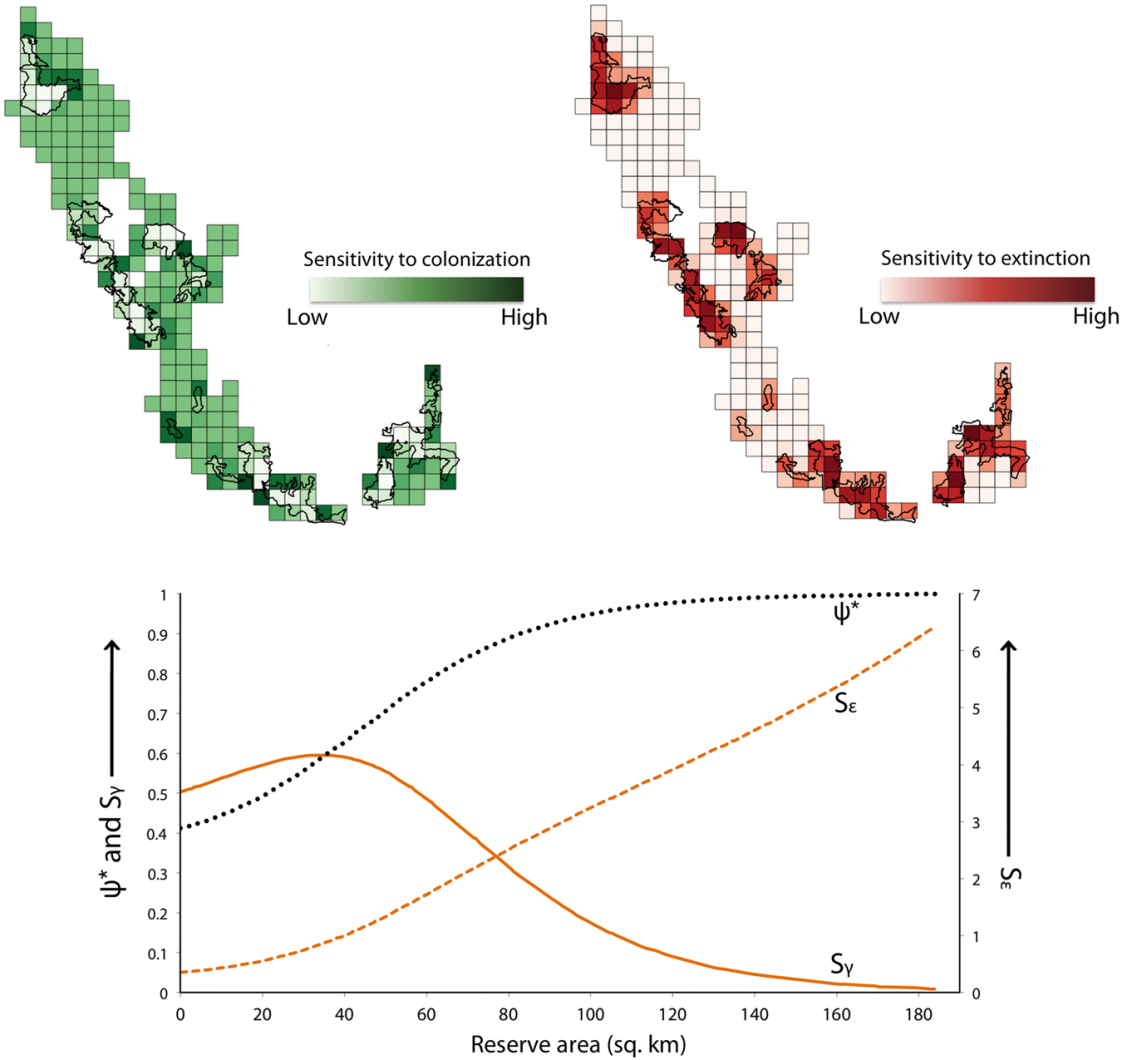
Sensitivity of equilibrium occupancy to colonization and local extinction. Top panel: Site-wise sensitivities of equilibrium occupancy (ψ^*^) to colonizations and local extinctions in Karnataka’s Western Ghats, from model where colonization γ and extinction ε were functions of reserve area. The darker shades represent sites where equilibrium occupancy is most sensitive to changes in colonizations and local extinctions. Bottom panel: ψ^*^, *S*_γ_ and *S*_ε_ expressed as functions of reserve area– values on primary y-axis correspond to ψ^*^ and *S*_γ_, values on secondary y-axis correspond to *S*_ε_.

**Figure 6:**
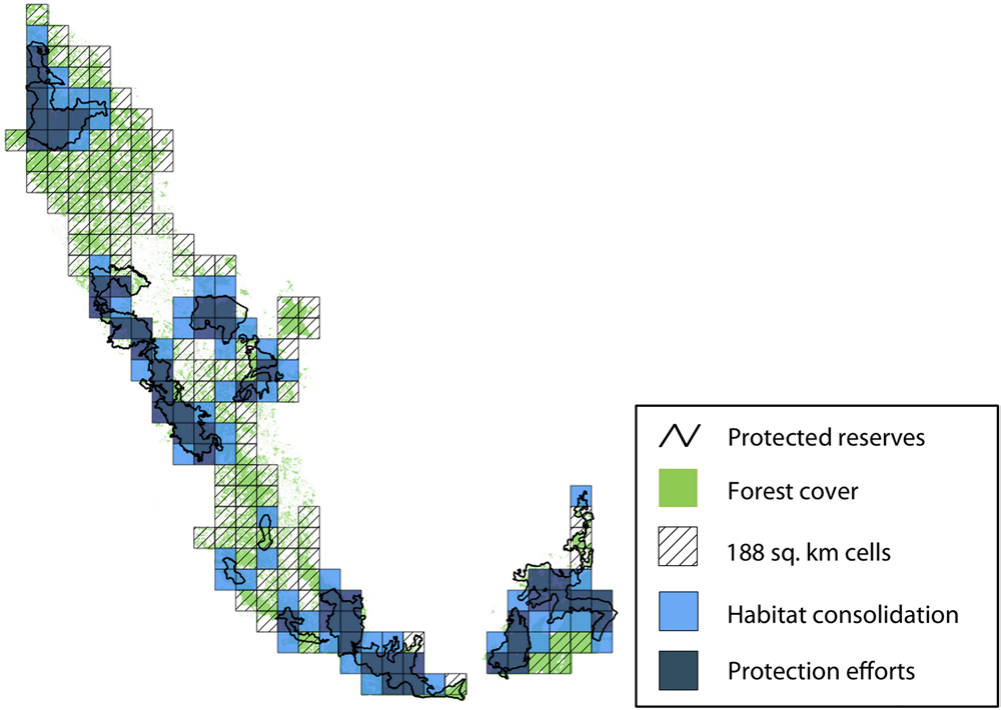
Prioritizing management actions for dhole conservation. Sites identified for prioritizing management interventions based on the top 25% quartile estimates of sensitivity: 49 sites to be targeted for habitat consolidation (high sensitivity to colonization) and 49 sites for sustaining or increasing protection efforts (high sensitivity to local extinction).

Based on the constrained model [γ(.), ε(.)], equilibrium occupancy for dholes in the landscape was ψ^*^ = 0.48 (corresponding *S*_γ_ = 0.89, *S*_ε_ = 0.83). We found that ψ^*^ for reserve sites was 0.71 (*S*_γ_ = 0.46, *S*_ε_ = 1.11), and for non-reserve sites ψ^*^ = 0.33 (*S*_γ_ = 0.58, *S*_ε_ = 0.29). Site-specific values of equilibrium occupancy, sensitivities to colonization and local extinction were calculated as functions of reserve area, as described in the Methods section. Spatial variation in sensitivity estimates are mapped in Fig. 5; the relationship between ψ^*^, *S*_ε_, *S*_γ_ and reserve area are also graphically presented in Fig. 5. Sites whose sensitivity values were in the upper 25% quartile included 49 cells each for colonization and local extinction (Fig. 6).

## Discussion

Determining the status and fate of metapopulations requires systematic, temporally replicated assessments of their structure and dynamics. We used information from the largest field-based study of dholes (in terms of spatial scale and rigor) to examine their spatio-temporal occupancy patterns, in a region that is critical for conservation of the species. Predicted dhole occupancy in Karnataka’s Western Ghats reduced from 62% in 2007 to 54% in 2015, suggesting a proportional decrease of 13%.

### Habitat quality in potential source sites

Establishment of protected reserves and efficient management practices have been pivotal in reversing population declines and preventing local extinctions of terrestrial vertebrates^36^. The reserves within our study region have had varying histories and intensities of protection^37^, and yet have succeeded in sustaining populations of many large mammal species. Most of these reserves were created for protection of tigers, but dholes may have co-benefited from such efforts^19^. We found that the average dhole occupancy for sites with reserves was higher than the overall average occupancy in the landscape during both the surveys (average occupancy for reserve sites was 68% in 2007 and 63% in 2015). Recent studies have additionally incorporated local demography of species to examine effects of population size (particularly in source patches) on the overall metapopulation dynamics (e.g., Sutherland, Elston & Lambin^32^). In the absence of robust methods to estimate dhole population sizes, the occupancy metric served as a reasonable surrogate for drawing inferences on the importance of reserves as population sources for this metapopulation.

Dholes are among the few large carnivore species that are both social, and restricted to forest habitats^14^. Determining ideal reserve sizes and carrying capacities of extant reserves for the species therefore presents a formidable challenge. Our results did not show a conclusive effect of reserve size on colonization or local extinction probabilities. Woodroffe and Ginsberg^38^ suggest ~700sq. km as an ideal minimum reserve size for viable dhole populations. However, their assessment does not take into account the potential competitive suppression by co-predators^37,39^ or the complex pack size dynamics in dholes^26,40^. In fact, the highly adaptable and dynamic nature of dhole packs^26^ is perhaps facilitating their persistence in Karnataka’s Western Ghats, where reserve sizes average at around 580sq. km (range = 111–1300sq. km). This further substantiates the critical need for maintaining inviolate reserves for dhole populations in the Ghats.

Anthropogenic factors pose the highest level of threats to large carnivore persistence globally^11^. Even with strict protection afforded to several reserves within our study region, most of these areas are not free of human intrusions and disturbances^23,41,42^. We chose relative abundance of livestock as a metric to characterize disturbance in Karnataka’s Ghats. As predicted, we found that livestock abundance to be negatively associated with dhole occupancy. The problem with livestock presence in forest habitats is two-fold– (1) large herds of cattle often accompanied by herders and their domestic dogs pose direct disturbance, since dholes generally avoid areas with high human activity^18,19,21^, and (2) livestock compete with wild herbivores for forage, and the subsequent livestock-mediated resource limitation causes depression of wild prey densities^24,43,44^, indirectly reducing the quality of potential dhole habitats. Our results therefore provide substantive evidence that reduction or total removal of livestock grazing pressures from protected reserves can be crucial for recovery and persistence of dhole populations.

### Local extinctions outside reserves

We found that the sites outside reserves generally had lower probabilities of dhole occupancy compared to estimates across the landscape (average occupancy in non-reserve sites was 53% in 2007 and 43% in 2015). Local extinction probabilities were also considerably higher outside reserves, primarily driven by loss of forest cover. Habitat loss can adversely impact source populations through geographic and demographic isolation^45,46^. Estimated colonization probability was higher than local extinction probability for dholes in the landscape; yet there was an overall decrease in occupancy from 2007 to 2015. This is because dynamic parameters are conditional upon the occupancy state of each site; colonizations can happen only in unoccupied sites and local extinctions only in occupied sites^30^. Considered together, net probability of local extinction was greater than net colonization probability. As with other important biodiversity areas in India, the Western Ghats landscape is currently in the midst of massive infrastructural development initiatives. Major highways have been upgraded by >1000 km and the village road network has expanded by ~5000 km. In Karnataka alone, 21 small hydropower dams have been commissioned (with 153 more planned or under various stages of construction) and 23 km of railway lines have been constructed (with 100 km more planned for construction) over the last decade. Such projects are often accompanied by collateral impacts on natural habitats through edge effects, degrading vegetation, change in land use, and expansion of human settlements into forest habitats^47–49^.

Forest loss was the principal driver of local extinctions (offset only by presence of reserves), yet a host of associated threats might be hampering recovery of dhole populations. India’s countryside and unprotected forests are inhabited by large populations of semi-feral, free ranging dogs^50^. Free-ranging dogs may be affecting dhole populations in three ways– (1) in areas where they spatially overlap, interference competition with packs of feral dogs may exclude dholes from certain locations and thereby reduce the total available habitat for dholes, (2) dogs are reservoirs of lethal and non-lethal disease pathogens^51^; interactions with dogs could result in spread of diseases and potentially bear negative consequences for dhole survival, and (3) local hunters sometimes use domestic dogs to chase dholes away from their kills in order to steal the carcasses^18^. Although we could not test the interaction between the two species, we suspect that dogs continue to pose a latent threat to dholes outside reserves. Future studies will need to explicitly examine the dynamics between the two species to fully understand the severity of this threat. We believe that the cumulative impacts of forest loss, infrastructure projects, and interactions with free-ranging dogs continue to impede range expansion of dholes in the landscape.

### Prioritizing management interventions

Colonization by dholes showed highly restrictive patterns, with colonized areas mostly clustered in and around reserves. But reserve presence alone did not affect colonization probabilities. This is perhaps because there were almost as many colonized sites in unprotected forests neighboring the reserves. Our treatment of the neighborhood effect therefore had the strongest (relatively, albeit uncertain) effect on patterns of colonization^33,52^. Furthermore, equilibrium occupancy in sites immediately surrounding wildlife reserves was most sensitive to colonizations (sites where reserve area ranged from 20–60sq. km). Some of these locations form critical links between important reserves. Increase in colonization in such sites would significantly increase the overall occupancy of dholes in the landscape (Fig. 5). The Indian government is currently in the process of denotifying or reducing the extent of ‘eco-sensitive zones’ that buffer the boundaries of reserves across the country. This would create hard habitat edges, reduce dhole habitats around reserves and further accelerate isolation of wildlife populations. We propose, therefore, that managers target such sites (Fig. 6) for habitat consolidation through increasing forest cover and promoting wildlife-friendly land-use practices. Making these sites more permeable for movement could facilitate colonization of dhole populations outside the reserve network and increase the extent and efficacy of sink habitats.

We identified 49 sites where equilibrium occupancy was most sensitive to local extinction, where extinction events would significantly decrease the overall dhole occupancy in the landscape. The dhole metapopulation would benefit if resources are invested towards improving the quality of habitat through increased protection efforts and recovery of prey populations in these sites. These locations include some reserves that currently support low prey densities and are afforded sub-optimal protection efforts (e.g., Kali, Kudremukh and Cauvery-MM Hills; Figs 1 and 6). Taken together, the twin effects of offsetting extinction probabilities through sustained/increased protection measures within select reserves, and expansion of forest cover or wildlife-permeable habitats around the reserves could hold the key for long-term conservation of the dhole metapopulation in Karnataka’s Ghats. While there have been recent efforts in this direction to consolidate forest lands and increase reserve sizes for tigers^53^, our assessment provides a more nuanced approach that is tailored specifically for conserving dholes.

### Implications for conservation

The Western Ghats landscape, in spite of its relatively high quality reserve network, does not seem adequate for conserving what may be the largest dhole metapopulation in the world. Since dholes are not prone to conflict with humans in this landscape^54^, nor hunted for meat or commercial trade of body parts^17^, conserving them does not entail other complexities as with most large carnivores. We caution however that dhole metapopulations in other parts of the country and elsewhere across dhole-range countries may face more imminent risks of local extinction. With just around 5% of India’s land area protected as wildlife reserves, and the reserves themselves being important for source populations, dholes may be facing a crisis situation. Increased protection in the Western Ghats’ reserves has enabled population recoveries of tigers and leopards^24,55^, but not for dhole populations. Although landscape-scale studies over multiple temporal replicates would reveal definitive trends in distribution patterns, our results still provide insights on factors that continue to hamper dhole population recoveries and range expansions.

Concurrent increases in global human and livestock populations, together with agricultural and infrastructure expansion, have rendered conserving carnivores a particularly difficult endeavor. Recognizing the importance of maintaining healthy metapopulations (rather than insular individual populations), studies are increasingly promoting a landscape-based approach to conserve wide-ranging carnivores threatened with endangerment^28,56,57^. Conservation initiatives for African wild dogs^58^, cougars *Puma concolor*^59^, and African lions *Panthera leo*^60^ have thus far demonstrated the utility of a metapopulation-centered strategy. Unfortunately, dhole distribution and range contraction assessments still rely expert opinion data and methods that ignore detection biases^12,17^, risking underestimation of areas with conservation potential. Here we present a cost-effective, statistically robust approach that may be adopted for periodic monitoring and status assessments of dholes across the species’ geographic range. We advocate for adopting similar strategies for assessment and management of dhole metapopulations in other regions to ensure their long-term persistence in heterogeneous landscapes.

## Supplementary information

Model selection table

## Data Availability

The data generated and analyzed during the study are available from co-author K.U.K (ukaranth@gmail.com) on reasonable request.
