# Supplementary material for: Insights from distribution dynamics inform strategies to conserve a dhole Cuon alpinus metapopulation in India
Source: Sci Rep. 2019 Feb 28;9:3081. doi: 10.1038/s41598-019-39293-0 (PMC6395595; doi:10.1038/s41598-019-39293-0)
Supplement: Supplementary file 1 — Model selection table [file 41598_2019_39293_MOESM1_ESM.docx]

**Title: Insights from distribution dynamics inform strategies to conserve a dhole *Cuon alpinus* metapopulation in India**

**Authors:** Arjun Srivathsa, K. Ullas Karanth, N. Samba Kumar, Madan K. Oli

**Table S1.** Full list of candidate covariate models used for modelling dhole occupancy in 2007 (ψ^1^), colonization (γ), local extinction (ε) and detection (*p_t_*) probabilities in the Western Ghats of Karnataka; cht- chital abundance; lvs- livestock abundance; nbr- neighborhood effect; fch- forest cover change; apr- all prey abundance; rpres- reserve presence; rarea- reserve area; ssn- season; K- number of parameters.

| **Model** | **Model description** | **AIC** | **ΔAIC** | **AIC weight** | **Model likelihood** | **K** | **Deviance** |
| --- | --- | --- | --- | --- | --- | --- | --- |
| M1 | ψ^1^(*cht+lvs*), θ^0^(.), θ^1^(.), γ(*nbr*), ε(*fch*), *p_t_*(*ssn+apr*) | 2318.25 | 0 | 0.24 | 1 | 14 | 2290.25 |
| M2 | ψ^1^(*cht+lvs*), θ^0^(.), θ^1^(.), γ(*nbr*), ε(*rpres*), *p_t_*(*ssn+apr*) | 2319.57 | 1.32 | 0.12 | 0.51 | 14 | 2291.57 |
| M3 | ψ^1^(*cht+lvs*), θ^0^(.), θ^1^(.), γ(.), ε(*fch*), *p_t_*(*ssn+apr*) | 2319.74 | 1.49 | 0.11 | 0.47 | 13 | 2293.74 |
| M4 | ψ^1^(*cht+lvs*), θ^0^(.), θ^1^(.), γ(*nbr+rpres*), ε(*fch*), *p_t_*(*ssn+apr*) | 2320.06 | 1.81 | 0.10 | 0.40 | 15 | 2290.06 |
| M5 | ψ^1^(*cht+lvs*), θ^0^(.), θ^1^(.), γ(*nbr+rarea*), ε(*fch*), *p_t_*(*ssn+apr*) | 2320.07 | 1.82 | 0.09 | 0.40 | 15 | 2290.07 |
| M6 | ψ^1^(*cht+lvs*), θ^0^(.), θ^1^(.), γ(.), ε(*rpres*), *p_t_*(*ssn+apr*) | 2320.54 | 2.29 | 0.08 | 0.32 | 13 | 2294.54 |
| M7 | ψ^1^(*cht+lvs*), θ^0^(.), θ^1^(.), γ(*nbr*), ε(*rarea*), *p_t_*(*ssn+apr*), | 2321.09 | 2.84 | 0.06 | 0.24 | 14 | 2293.09 |
| M8 | ψ^1^(*cht+lvs*), θ^0^(.), θ^1^(.), γ(*fch*), ε(*fch*), *p_t_*(*ssn+apr*) | 2321.28 | 3.03 | 0.05 | 0.22 | 14 | 2293.28 |
| M9 | ψ^1^(*cht+lvs*), θ^0^(.), θ^1^(.), γ(*nbr+rpres*), ε(*rpres*), *p_t_*(*ssn+apr*) | 2321.56 | 3.31 | 0.05 | 0.19 | 15 | 2291.56 |
| M10 | ψ^1^(*cht+lvs*), θ^0^(.), θ^1^(.), γ(*rpres*), ε(*rpres*), *p_t_*(*ssn+apr*) | 2322.48 | 4.23 | 0.03 | 0.12 | 14 | 2294.48 |
| M11 | ψ^1^(*cht+lvs*), θ^0^(.), θ^1^(.), γ(*nbr)*, ε(*fch+rarea*), *p_t_*(*ssn+apr*) | 2322.59 | 4.34 | 0.03 | 0.11 | 15 | 2292.59 |
| M12 | ψ^1^(*cht+lvs*), θ^0^(.), θ^1^(.), γ(*rarea*), ε(*rarea*), *p_t_*(*ssn+apr*) | 2324.01 | 5.76 | 0.01 | 0.06 | 14 | 2296.01 |
| M13 | ψ^1^(*cht+lvs*), θ^0^(.), θ^1^(.), γ(.), ε(*fch+rarea*), *p_t_*(*ssn+apr*) | 2324.30 | 6.05 | 0.01 | 0.05 | 14 | 2296.30 |
| M14 | ψ^1^(*cht+lvs*), θ^0^(.), θ^1^(.), γ(*nbr*), ε(*nbr*), *p_t_*(*ssn+apr*) | 2326.03 | 7.78 | 0.00 | 0.02 | 14 | 2298.03 |
| M15 | ψ^1^(*cht+lvs*), θ^0^(.), θ^1^(.), γ(.), ε(.), *p_t_*(*ssn+apr*) | 2327.16 | 8.91 | 0.00 | 0.01 | 12 | 2303.16 |
| M16 | ψ^1^(*cht+lvs*), θ^0^(.), θ^1^(.), γ(.), ε(*nbr*), *p_t_*(*ssn+apr*) | 2327.51 | 9.26 | 0.00 | 0.01 | 13 | 2301.51 |
| M17 | ψ^1^(.), θ^0^(.), θ^1^(.), γ(.), ε(.), *p_t_*(*ssn+apr*) | 2340.85 | 22.6 | 0 | 0 | 10 | 2320.85 |
| M18 | ψ^1^(.), θ^0^(*ssn*), θ^1^(*ssn*), γ(.), ε(.), *p_t_*(*ssn+apr*) | 2344.51 | 26.26 | 0 | 0 | 12 | 2320.51 |
| M19 | ψ^1^(.), θ^0^(.), θ^1^(.), γ(.), ε(.), *p_t_*(*apr*), | 2348.04 | 29.79 | 0 | 0 | 8 | 2332.04 |
| M20 | ψ^1^(.), θ^0^(.), θ^1^(.), γ(.), ε(.), *p_t_*(.) | 2363.71 | 45.46 | 0 | 0 | 7 | 2349.71 |
| M21 | ψ^1^(.), θ^0^(.), θ^1^(.), γ(.), ε(.), *p_t_*(*ssn*) | 2365.33 | 47.08 | 0 | 0 | 8 | 2349.33 |
